# Investigation of illicit pregabalin in seized samples from Saudi Arabia

**DOI:** 10.3389/fchem.2025.1594567

**Published:** 2025-07-23

**Authors:** Fatimah M. Alamri, Sultan K. Alshmmari, Monerah A. Altamimy, Ibrahim A. Al Othaim, Yahya M. Alshehri, Rayed M. Alafraa, Ahmed D. Almalki, Turki A. Alkhalifah, Taher Sahlabji, Abubakr M. Idris, Haitham Al-Hamoud, Yahya F. Jamous, Fahad S. Aldawasri

**Affiliations:** ^1^ Department of Chemistry, College of Science, King Khalid University (KKU), Abha, Saudi Arabia; ^2^ Department of Chemistry, College of Science, University of Bisha (UB), Bisha, Saudi Arabia; ^3^ Saudi Food & Drug Authority (SFDA), Riyadh, Saudi Arabia; ^4^ General Directorate of Narcotics Control, Ministry of Interior (MOI), Riyadh, Saudi Arabia; ^5^ Research Center for Advanced Materials Science (RCAMS), King Khalid University, Abha, Saudi Arabia; ^6^ Institute of Refining and Petrochemical Technologies, Energy and Industry Sector, King Abdulaziz City for Science and Technology (KACST), Riyadh, Saudi Arabia; ^7^ Institute of Health Technologies and Preventive Medicine—Health Sector, King Abdulaziz City for Science and Technology (KACST), Riyadh, Saudi Arabia

**Keywords:** pregabalin, forensic analysis, abused drugs, seized samples, counterfeit drugs, adulterants, ultra-performance liquid chromatography, tandem mass spectrometry

## Abstract

**Introduction:**

Pregabalin (PGL) is a medication that is prescribed for controlling specific neurological-related symptoms. Due to its abuse in multiple countries, PGL has been classified as a controlled substance by authorities, including the Saudi Food and Drug Authority (SFDA).

**Methods:**

This study developed a validated ultra-performance liquid chromatography-photodiode array detector (UPLC-PDA) method to quantify PGL in 40 seized samples (35 capsules, 5 powders). A complementary liquid chromatography-tandem mass spectrometry (LC-MS/MS) method was used to detect potential adulterants.

**Results:**

The UPLC-PDA method demonstrated linearity (r = 0.9973) for PGL quantification (0.50–3.00 mg/mL), with an accuracy of 96%–102%. The RSD% values were 0.63% and 1.03% for intra-day and inter-day precision, respectively. Analysis of the five powder samples revealed a relative inconsistency in PGL content (107.91%–114.55%). Moreover, it showed higher variability in PGL content (RSD 1.16%–5.30%), suggesting possible adulteration or poor manufacturing. Furthermore, the results of the nuclear magnetic resonance (NMR) showed an acceptable purity for the powder samples. On the other hand, among 35 capsules, 5 (14.29%) exceeded pharmacopeial limits (95%–105% PGL content), while 6 (17.14%) contained <95% PGL.

**Discussion:**

These results demonstrate significant variability in PGL content and the presence of adulterants, underscoring the need for robust analytical methods in forensic chemistry. Furthermore, the LC-MS/MS method detected adulteration of PGL with codeine, paracetamol, and gabapentin in 2.9% of the analyzed capsules, suggesting custom mixing by perpetrators. In general, 31.43% of these samples failed to meet quality standards and contained substances beyond declared contents that posed toxicity risks, revealing inadequacies in illicit drug production and circulation. The UPLC-PDA method offers a rapid, validated approach for PGL quantification, while LC-MS/MS enhances adulterant detection, supporting forensic and quality control applications.

## 1 Introduction

**FIGURE 1:**
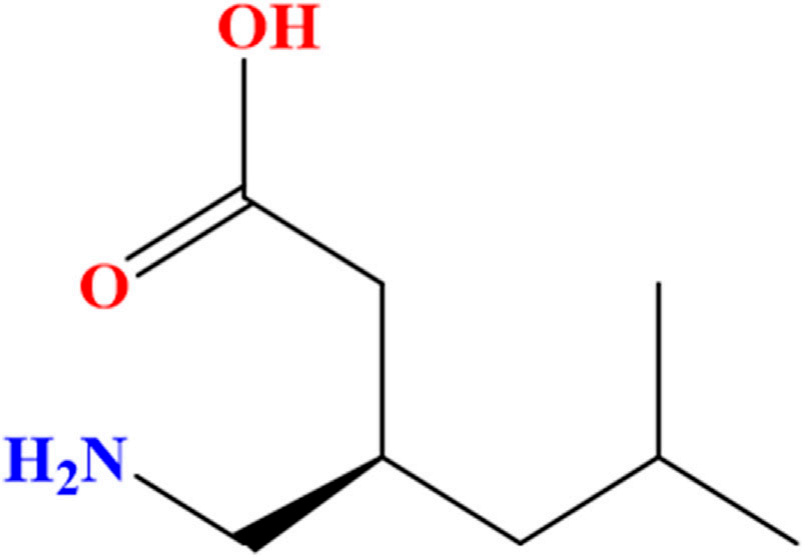
Chemical structure of the (S)-enantiomer of pregabalin [(S)-(+)-3-(aminomethyl)-5-methylhexanoic acid].

Pregabalin (PGL), a gabapentinoid, is an antiepileptic and analgesic that modulates voltage-gated calcium channels to treat neuropathic pain and epilepsy (Kriikku and Ojanperä, 2021). It is chemically named (*S*)-(+)-3-(aminomethyl)-5-methylhexanoic acid (C_8_H_17_NO_2_) (Figure 1) with a molecular weight of 159.23 g/mol. PGL has a white, crystalline solid form (Toth, 2014). In December 2004, the Food and Drug Agency (FDA) in the United States of America (United States) approved PGL for the treatment of neuropathic pain associated with diabetic peripheral neuropathy and postherpetic neuralgia. Since then, PGL has been approved in the United States and Europe for the treatment of these symptoms (Tassone et al., 2007). In Europe, PGL has also received regulatory approval to treat a variety of clinical conditions, as it demonstrates a wide range of therapeutic utility. Subsequently, PGL has also been recommended as a medication for seizures, neuropathic pain states, and anxiety-related disorders. PGL serves as a valuable adjunctive option for individuals with partial epilepsy as well (Asomaning et al., 2016). Furthermore, it works by slowing down nerve activity in the brain and spinal cord. On the contrary, PGL commonly causes dizziness, drowsiness, blurred vision, and dry mouth (Mutalabisin et al., 2021). It can produce euphoria and relaxation (Kriikku and Ojanperä, 2021; Evoy et al., 2017), increasing its significant risks of abuse, which include respiratory depression, dependence, and, in rare cases, severe allergic reactions. Overdose may cause seizures, coma, or dangerously low blood pressure (Alelwani et al., 2021).

PGL has emerged as a drug of abuse owning to several factors such as its intoxicating effects, potential to enhance the euphoric and sedative effects of other CNS depressants (e.g., opioids, benzodiazepines), ability to self-treat pain/withdrawal symptoms, accessibility as a generic medication and its association with opioid abuse. Moreover, the prevalence of non-medical use of PGL is expected due to its affordable price (Buttram and Kurtz, 2020). PGL was among the top-selling medications globally in 2017. During the year 2018, in England, more than 14 million PGL prescriptions were issued. This was probably driven by refraining from opioid analgesics. In April 2019, the United Kingdom reported significant gabapentinoid-related deaths due to misuse, highlighting their abuse potential (Mathieson et al., 2020). In this context, it is worth mentioning the definition of the term abuse, which is defined as “the substance that is used for nontherapeutic purposes to obtain psychotropic (e.g., euphoric, sedative, or anxiolytic) effects” (Smith et al., 2013).

Based on the above-mentioned data, there are growing concerns about the abuse, addiction, intoxication, and even fatalities associated with PGL. Accordingly, PGL was classified in December 2017 by the Saudi Food and Drug Authority (SFDA) as one of the controlled prescription drugs that has been facing issues of abuse and illegal trafficking (Althunian et al., 2022).

It is noteworthy that PGL has been considered subject to abuse when formulated with various substances. Several samples, which contained numerous substances and manufactured by unauthorized factories for non-medical purposes, were seized in some countries. Common compounds detected alongside PGL included cannabis products, opioids (e.g., morphine, hydromorphone and codeine), semi-synthetic analogues (e.g., hydrocodone), and over-the-counter medications (e.g., paracetamol and naproxen) (Teker et al., 2024). In connection to this, health risks linked to the abuse of PGL with other compounds are considered serious. As PGL is known to depress the Central Nervous System (CNS), its use with other CNS depressants significantly elevates the danger of severe toxicity and mortality. When combined with opioids, cannabis products, and other depressing agents, PGL loses its therapeutic efficacy and considerably enhances the risk of severe adverse effects or potentially fatal outcomes (Elliott et al., 2017). Hence, developing selective analytical methods for PGL in the presence of various additives is highly desirable to be applied at forensic, toxicological, and pharmaceutical laboratories.

It is well-known that pharmaceutical analysis relies heavily on liquid chromatography (LC). Unfortunately, direct LC analysis of PGL faces some challenges, including a lack of chromophores, interference with excipients that absorb at similar wavelengths, and instability due to oxidative degradation (Mutalabisin et al., 2021; Kritikos et al., 2022). As a result, identification and quantification of PGL are considered a hurdle during initial routine procedures for laboratory screening. These analytical challenges necessitate the development of more efficient assay methods, particularly for confirmation analysis of PGL when it is suspected to contribute to adverse clinical presentations or toxicities (Mutalabisin et al., 2021). In this context, a limited number of analytical methods were developed for PGL quantification in various sample matrices for clinical and forensic applications. LC with mass spectrometric detections (MS), including tandem MS and TOF-MS methods, are dominant in the literature. Through these methods, PGL levels were examined in various human samples, including blood (Mata and Davis, 2022; Nahar et al., 2017; De La Vega et al., 2019), plasma (Almalki et al., 2021), urine (Mata and Davis, 2022; Almalki et al., 2021; Wang and Xu, 2019; Pelander et al., 2010), saliva (Wang and Xu, 2019), gastric contents (Mata and Davis, 2022), vitreous humor (Pelander et al., 2010), liver and brain tissues (Mata and Davis, 2022). Environmental analysis of urban sewage samples for determination of PGL was also reported using LC-MS/MS (Gurke et al., 2015). In addition, high-performance liquid chromatography (HPLC) with fluorescence and UV detection have been reported for PGL assay in human plasma for therapeutic drug monitoring (Martinc et al., 2014) as well as bulk and tablet formulations for forensic investigations (Vinutha Ko et al., 2017; Khanage et al., 2014). Recent studies have well-documented PGL misuse, particularly among polydrug users and opioid-dependent individuals (Kriikku and Ojanperä, 2021). To that end, some regulatory warnings and mortality data have further confirmed its abuse potential (Mathieson et al., 2020; Elliott et al., 2017).

This study provides analytical data on locally seized PGL samples. Furthermore, the study aims to identify PGL contents and purity levels along with potential adulteration. The study combines two analytical methods, i.e., ultra-performance liquid chromatography with photodiode array detector (UPLC-PDA) and LC-MS/MS. The HPLC-UV method recommended by the British Pharmacopoeia (BP) (British Pharmacopoeia, 2022) was upgraded to UPLC-PDA and then validated. Then, the new method was applied for PGL assay in capsule and powder samples while an LC-MS/MS method was performed for confirmation of the adulterated seized samples.

## 2 Materials and methods

### 2.1 Sampling

**FIGURE 2:**
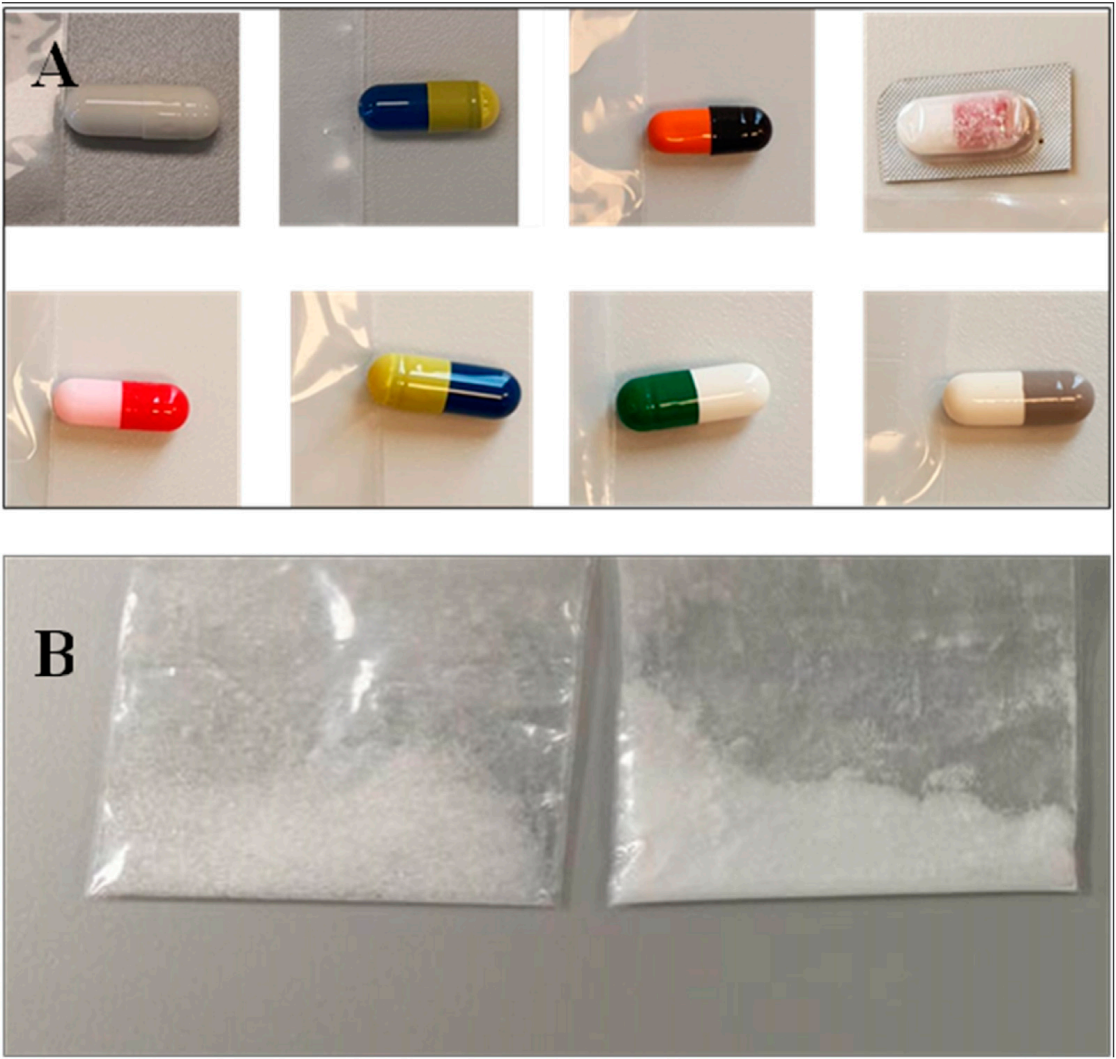
Representative seized pregabalin samples showing physical variability: **(A)** Capsules with differing shapes/colours where be difficult to trace, and **(B)** Powder formulations with inconsistent solid state.

The forensic samples examined in the current study were seized by the General Directorate of Narcotics Control (GDNC), Saudi Arabia. The seized samples consisted of 35 capsules and 5 powder forms suspected to contain PGL. The capsules varied in shape, colour, and form, while the powders were likely intended for clandestine capsule production (Figure 2). All samples were handled under proper chain-of-custody protocols and stored at room temperature in a dark place to preserve sample integrity for analytical testing.

### 2.2 Chemicals and reagents

PGL reference standard (purity ≥99.90%) was obtained from HPC Standards GmbH (Germany). HPLC-grade acetonitrile, formic acid, and ammonium formate were purchased from Sigma-Aldrich (Germany), while potassium dihydrogen orthophosphate and sodium hydroxide were sourced from Fisher Scientific (United Kingdom). Purified water was prepared using a Duo™ water purification system from Avidity Science™ (United States).

### 2.3 Instrumentation

A Waters^®^ ACQUITY UPLC-PDA system was used for separation, equipped with a Phenomenex C18 column (2.1 × 50 mm, 1.8 µm). For mass spectrometry investigation, a Thermo Scientific^®^ LC-MS/MS system was used. Structural nuclear magnetic resonance (NMR) elucidation was performed by JEOL JNM-ECA600II NMR (deuterium oxide (D_2_O) solvent, tetramethylsilane (TMS) as a reference).

### 2.4 Sample preparation

Powder samples (∼20 mg) were dissolved in 10 mL of water: acetonitrile (90:10), while capsule contents were extracted in 50 mL of the same solvent. All samples were agitated for 10 min (1,000 rpm), filtered through a 0.22 μm nylon syringe filter, and analyzed in triplicate for quality control.

The NMR sample was prepared by accurately dissolving 5 mg of PGL powder sample in 0.5 mL of D_2_O without filtration. The resulting solution was transferred into a 5 mm NMR tube for spectral acquisition under standard operating conditions.

### 2.5 Standard solution preparation

The stock solution of PGL (5.0 mg/mL) was prepared by dissolving appropriate amounts in acetonitrile:water (10:90) solution. Working solutions of PGL were prepared from the stock solution by further dilution with acetonitrile:water (10:90) solution at concentrations of 0.50, 0.75, 1.00, 1.25, 1.50, 2.00, and 3.00 mg/mL.

### 2.6 Chromatographic system conditions

For the UPLC-PDA method, the separation was achieved into a Phenomenex 2.1 mm ID, 50 mm C18 column with particle size 1.8 µm. A gradient elution was applied using a mobile phase composed of Solution A and Solution B in a 9:1 ratio, respectively. The composition was varied over time to effectively separate PGL from any potential impurities or adulterants (Table 1). While solution-B was acetonitrile, solution-A was potassium dihydrogen orthophosphate (0.272% w/v) adjusted at a pH of 5.9 with 1 M sodium hydroxide. The mobile phase was delivered at a flow rate of 0.5 mL/min with a total runtime of 8.6 min. The column temperature was set at 40^0^ C. The sample volume was adjusted at 3 µL. The PDA detector was set at 210 nm wavelength.

**TABLE 1:** The gradient elution protocol for the UPLC-PDA method.

Time (min)	Mobile phase a (%v/v)	Mobile phase B (%v/v)
0	100	0
1.1	100	0
4.4	60	40
5.6	60	40
6.6	100	0
8.6	100	0

For the LC-MS/MS method, an isocratic elution with two mobile phases was applied. Mobile phase C was composed of acetonitrile with 0.1% formic acid and mobile phase D was composed of 5 mmol of ammonium format in 0.1% (v/v) formic acid. Mobile phases C and D were used at a constant ratio (20:80) during the analysis. The method was conducted in the positive ionization mode. The specific mass transitions were monitored to detect the target compounds (Table 2).

**TABLE 2:** The composition of mobile phase elution of LC-MS/MS.

Time (min)	Flow (mL/min)	Mobile phase C (%v/v)	Mobile phase D (%v/v)	Curve
0	0.5	20	80	5
2	0.5	20	80	5
2	Stop Run			

### 2.7 Method validation

The HPLC assay method for PGL, which was recommended by the British Pharmacopoeia (BP) (British Pharmacopoeia, 2022), was upgraded to a UPLC-PDA method. The linearity, accuracy, precision, limits of detection (LOD) and quantification (LOQ) were validated according to International Council for Harmonisation (ICH) guidelines (Abraham et al., 2010).

The calibration curve was prepared using seven calibrators over a concentration range of 0.50–3.00 mg/mL for PGL and injected into the UPLC system, with each concentration analyzed in triplicate (n = 3). Linear regression was used to plot peak area versus concentration. The linear range was assessed visually from the calibration curve and by calculating the correlation coefficient (r).

Specificity was assessed by injecting blank and placebo solutions, ensuring no interfering peaks appeared at the retention time of the target analyte. Carry-over was evaluated by injecting blank solvent (acetonitrile: water, 10:90) immediately following the highest calibration standard (3.00 mg/mL), with acceptance criteria requiring signal in blanks to be <20% of the LOQ for specificity and ≤5% of the upper calibration limit for carry-over.

The accuracy was calculated using Equation 1. The precision was determined as the relative standard deviation (RSD%) of replicate measurements at various concentrations*,* calculated using Equation 2. Intraday precision was assessed via three replicates for each of three different concentrations (0.75, 1.25, and 2.00 mg/mL) in one run. Inter-day studies analyzed three replicates per concentration of (0.75, 1.25, and 2.00 mg/mL) across six consecutive days.

$$Accuracy\;\left(\%\right)=\frac{Calculate\;concentration}{Nominal\;Concentration}\;X\;100$$ (1)

$$RSD\%=\frac{Standard\;deviation\;of\;peak\;areas\;\left(SD\right)}{Mean\;peak\;areas}\;X\;100$$ (2)

The LOD and LOQ were established based on the calibration curve parameters by using the standard deviation of response (SD) and slope (S) obtained from linear regression of the seven-point calibration curve as per Equations 3, 4, according to ICH Q2 (R1).

$$LOD=3.3\left(SD/S\right)$$ (3)

$$LOQ=10\left(SD/S\right)$$ (4)

Statistical analyses were performed using Empower™ (Waters) and XCalibur™ (Thermo) software, with supplementary calculations conducted in Microsoft Excel.

## 3 Results and discussion

### 3.1 UPLC-PDA Method validation

The upgrading of the PGL assay method from HPLC to UPLC resulted in significant reductions in analysis run time from 17.0 min down to 8.6 min. The sampling frequency was improved from 2 samples/h using the HPLC method to 4 samples/h using the UPLC method; an advantage resulted in better laboratory productivity. It is also known that sample throughput is a critical aspect of forensic analysis that requires rapid analysis of urgent samples. Additionally, the use of UPLC method reduces chemical reagent consumption and waste generation; an issue that achieves a less environmentally harmful method. These benefits were obtained by decreasing the mobile phase flow rate from 1 mL/min using the HPLC method to 0.5 mL/min using the UPLC method. The sample injection volume was also reduced from 20 μL to just 3 μL. As a result, the total waste volume generated per sample is reduced significantly from 27.020 mL to 6.803 mL, representing a 74.8% reduction in waste volume. On the other hand, the UPLC system achieved enhanced separation efficiency at the optimized flow rate (0.5 mL/min), reducing the analysis time by 49.4% compared to HPLC (1.0 mL/min).

### 3.2 Analysis of powder samples

**FIGURE 3:**
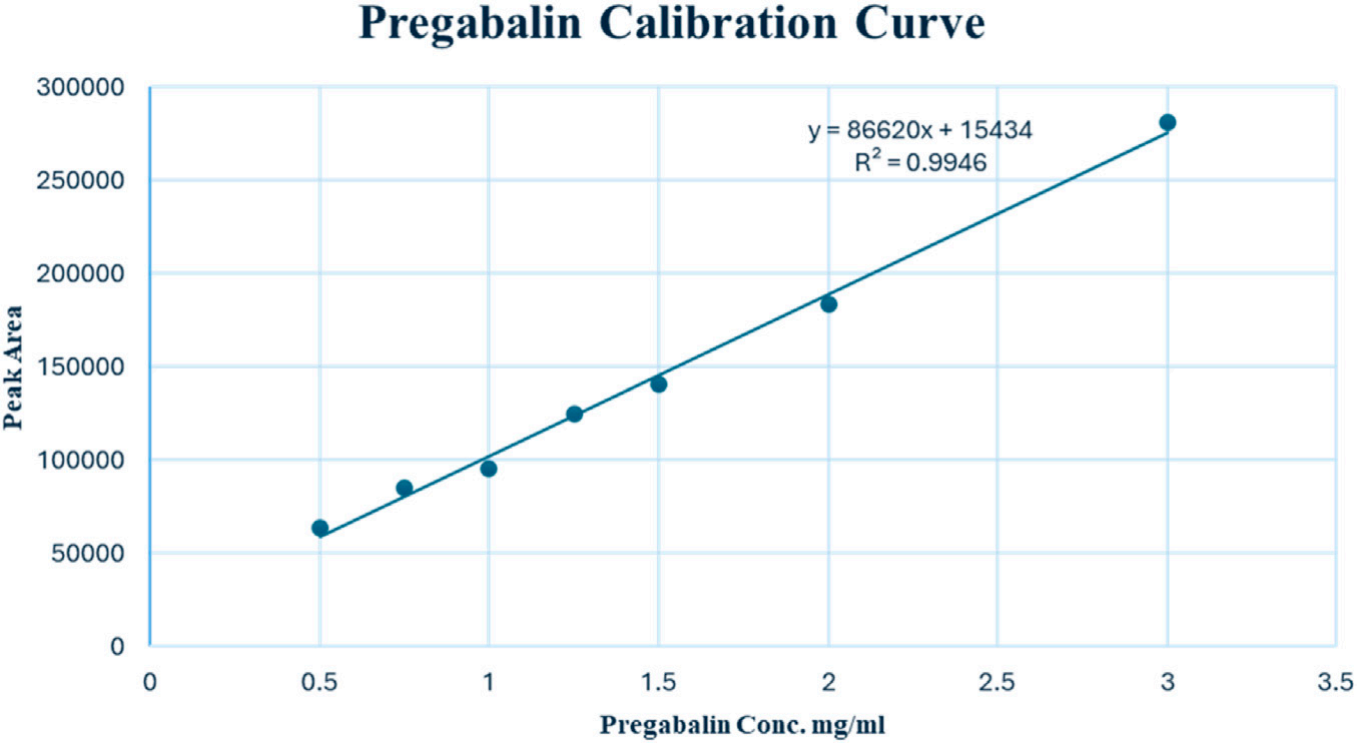
Calibration curve of pregabalin (0.50–3.00 mg/mL) by UPLC-PDA.

The linearity of the modified UPLC method was evaluated by analysing a series of calibration standards. The method was found to be linear (Figure 3), with a correlation coefficient (r) of 0.9973, in the concentration range of 0.50–3.00 mg/mL for PGL. Using the linear least square regression method of the seven-point calibration curve (r = 0.9973), the slope and intercept for PGL were found to be 86,620 and 15,433, respectively. Despite a narrow linear range, the calibration features a high slope value, indicating a good response to slight changes in the concentration. The RSD% value of repeated measurements was found to be 0.628%, suggesting good repeatability of the UPLC method. It is reported that the typically acceptable is <2% (United States Pharmacopeia, 2023). Notably, the SD of the slope was 2,787, while the SD of the intercept was 4,547, reflecting acceptable variability in the slope and intercept calculations. In addition, the standard error of the estimate was 5,807, being relatively low, indicating the closeness of the data points to the calibration curve. This confirms a good fit of the linear regression model to the calibration data.

The LOD and LOQ values based on residual SD of the regression line were 0.23 and 0.69 mg/mL, respectively. Both values of LOD and LOQ are suitable for pharmaceutical analysis as PGL is a major component in seized samples as well as pharmaceutical formulations.

The method validation demonstrated excellent specificity, with no detectable interfering peaks at the retention time of PGL (0.36 min) in the blank and placebo solutions. All blank injections showed signals below 20% of the LOQ (0.69 mg/mL), confirming the method’s selectivity for pregabalin detection. Carry-over evaluation revealed minimal residual signal (<5% of the upper calibration limit) in blank solvent injections following the highest standard (3.00 mg/mL), indicating acceptable system cleanliness for high-throughput analysis. These results confirm the reliability of the method for analyzing seized samples, as any potential matrix effects or carry-over contamination would not significantly impact the quantification accuracy.

For system suitability, as shown in Table 3, six independent preparations of the PGL reference standard (2.00 mg/mL) were subjected to the modified UPLC method on day one. System suitability was confirmed by meeting the following acceptance criteria: peak area RSD% (<2%), retention time variation (<2%), theoretical plates (>2000), and peak asymmetry (0.9–1.5). Evidently, the observed RSD% of peak areas was 1.16%, demonstrating excellent intraday precision. Moreover, the RSD% of the peak areas was found to be 1.16%. This result shows good intraday precision. The accuracy ranged between 96% and 102% for PGL analysis. Collectively, the RSD% values were 0.63% and 1.03% for intra-day and inter-day precision, respectively, demonstrating excellent performance for both repeatability and reproducibility of the analytical method over the tested concentration range.

**TABLE 3:** System suitability of six independent preparations of PGL reference standard (2 mg/mL) for UPLC-PDA method.

Concentration of PGL reference standard (mg/mL)	Replicates	Mean area	SD	RSD%
2	6	184,747	2,142.5	1.16%

### 3.3 Analysis of capsule samples

The powder samples for raw material were visually investigated which demonstrated irrational results. A noticeable discrepancy was observed between different powder samples in terms of crystal size and shape. Irregular crystal structures were noticed compared to the well-defined structures typically expected for pure PGL. This observation might indicate a lack of homogeneity and potentially confirms poor manufacturing practices.

**FIGURE 4:**
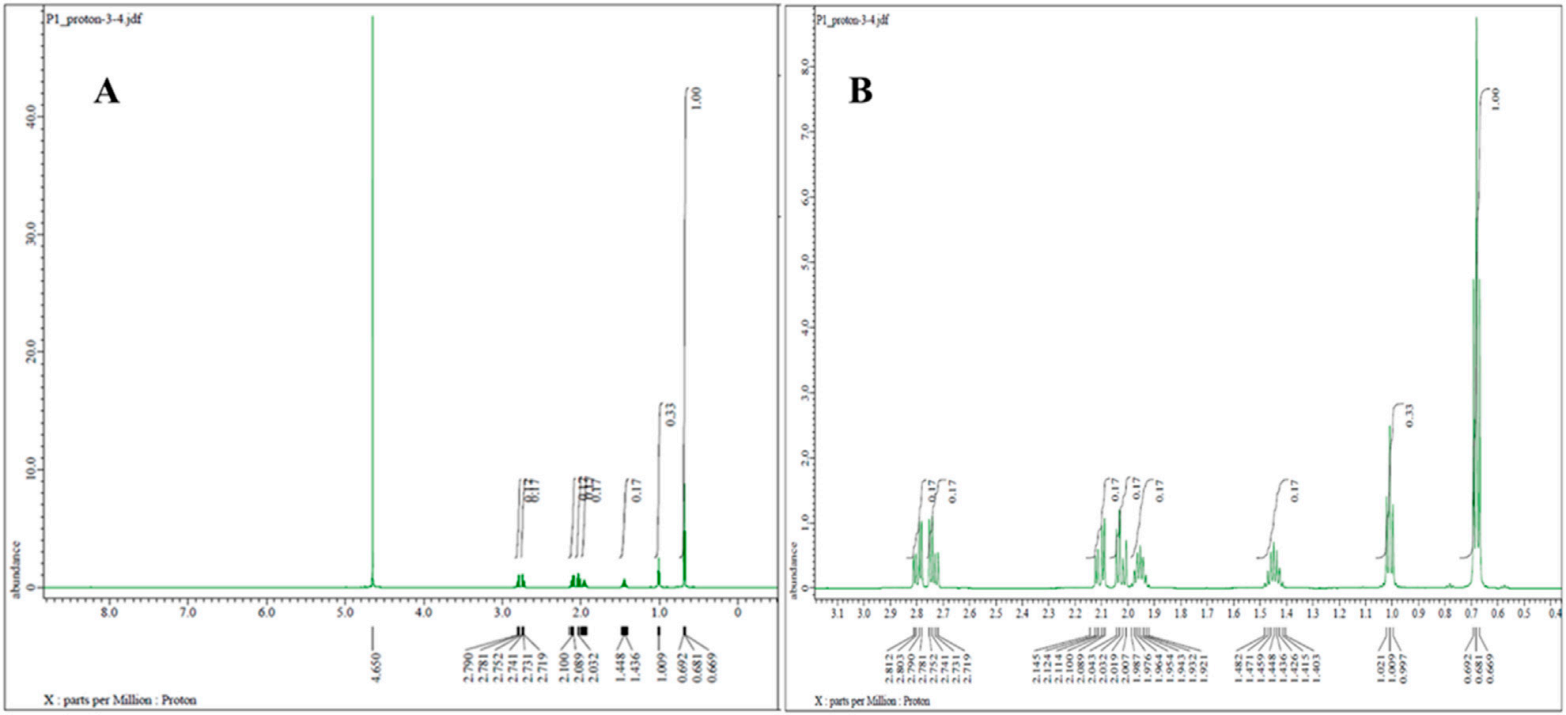
^1^H NMR spectrum (600 MHz, D_2_O) of pregabalin powder representative sample **(A)** with zoom-in from 1 to 3 ppm **(B)**, showing characteristic proton signals of pregabalin.

**FIGURE 5:**
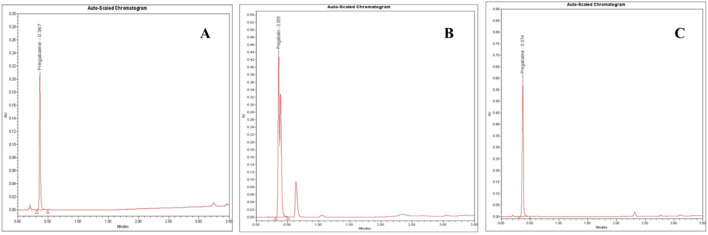
UPLC-PDA chromatograms of: **(A)** pregabalin standard (5.0 mg/mL), **(B)** an adulterated seized capsule sample showing pregabalin (Rt = 0.36 min) and unidentified impurities and **(C)** a seized pregabalin capsule sample. Chromatographic conditions: C18 column (2.1 mm ID, 50 mm, particle size 1.8 µm), mobile phase: buffer (pH 5.9): acetonitrile (9:1 ratio), flow rate 0.5 mL/min, column temperature at 40 °C and UV detection at 210 nm.

Proton NMR analysis for the five samples of the powder forms revealed comparable results in which all 14 alkyl protons in the PGL structure (Figure 1) were demonstrated in well-defined signals (Figure 4). These characterizations were in agreement with a typical NMR spectrum for PGL (Komisarek et al., 2022). This finding may illustrate acceptable purity in terms of PGL itself and not necessarily other adulterants or impurities. On the other side, the UPLC method was applied to the powder samples for quantitative analysis of PGL. As shown in Table 4, the analytical results of the powder samples for PGL showed higher concentrations than the reference standard (Figure 5A), suggesting the powders contained impurities/additional compounds other than the expected PGL. Notably, the United States Pharmacopeia (USP) recommended the acceptable range for PGL assay is 98%–102% on a dried basis. PGL concentrations above the recommended range suggest that the powder samples likely contain other substances.

**TABLE 4:** UPLC-PDA analysis of three independent preparations of each seized pregabalin powder sample.

Powder samples	Measured conc. (mg/mL)	PGL %	Mean	SD	RSD%
1P-1	2.051	109.66	114.55	6.07	5.30
1P-2	2.099	112.66			
1P-3	2.162	121.34			
2P-1	2.046	109.12	107.91	1.25	1.16
2P-2	2.044	107.98			
2P-3	2.023	106.63			
3P-1	2.103	111.01	111.84	2.19	1.96
3P-2	2.102	110.18			
3P-3	2.097	114.33			
4P-1	2.100	114.89	112.88	1.80	1.59
4P-2	2.063	111.44			
4P-3	2.104	112.32			
5P-1	2.09	109.92	111.25	1.95	1.75
5P-2	2.038	113.49			
5P-3	2.097	110.33			

Further contributing factors included potential adulteration, as additional chromatographic peaks beyond PGL implied other constituents (Figure 5B). Issues such as moisture absorption or degradation from inadequate storage conditions over time could also have unpredictably skewed measured values where PGL is susceptible to various degradation pathways, including hydrolysis, oxidation, thermal degradation, and photodegradation, which may contribute to the observed purity variations in seized samples. Hydrolysis can occur under acidic and alkaline conditions, causing the ester bond in PGL to cleave, forming the acid and alcohol derivatives. In addition, thermal degradation results in the loss of a water molecule and oxidation in the presence of oxidizing substances like hydrogen peroxide and oxygen to form N-(3-carbamoylpropyl) isobutyric acid. This thermal degradant has a similar structure and could co-elute with PGL peak. Forced degradation studies, as per ICH guidelines, observed major degradation under basic hydrolysis and oxidation conditions, forming ten unknown oxidative impurities characterized using LC-MS, HR-MS and NMR. The proposed oxidative degradation pathway involves sequential formation of mono-hydroperoxide, cyclized hydroperoxide, dihydroperoxide and hydroxy derivatives that eventually degrade into multiple products (Vukkum et al., 2015). Issues related to purity, consistency, production quality control, and stability prevented the analytical results of the powder samples from aligning with the established reference values. A study (Sripathi et al., 2010) highlighted the importance of impurity characterization in manufacturing PGL pharmaceutical formulation to meet regulatory standards, safety and efficacy requirements. This recommendation aids in the optimization of manufacturing methods and reduces the formation of undesirable impurities. Another study reported that variations in the manufacturing process could result in significant differences in the uniformity of PGL formulations, which may have a reverse effect on patient safety (Gujral et al., 2009).

### 3.4 Method upgrading from HPLC to UPLC

The seized PGL capsule samples (n = 35) were examined for shape, size, and colour. These parameters provide initial clues about potential sources. In the current study, the investigation showed different shapes, colours, and forms. The comparison of the physical attributes of seized capsules to licensed product profiles helped in tracing some capsules back to major manufacturing facilities. The traceability of physical properties suggests that 21 seized samples were from four facilities, while 14 samples were difficult to trace. The presence of untraceable capsules suggests that they are counterfeit drugs randomly filled by unqualified illegal operators rather than legitimate pharmaceutical companies.

**FIGURE 6:**
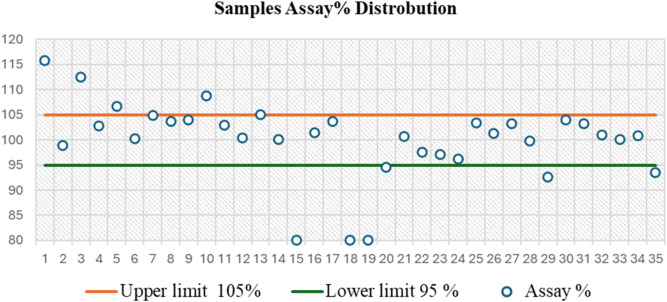
Control chart based on assay distribution between British Pharmacopoeia (BP) specifications (95%–105%) for the analysis of 35 capsule samples, as determined by the UPLC-PDA method, showing that 31.43% of these samples failed to meet the BP limit.

On the other side, the results of the PGL assay for the seized capsule samples (Figure 5C) using the UPLC-PDA method are shown in Table 5. The results revealed that five capsule samples containing PGL exceeded the range of 95%–105%, which is recommended by the BP. While most samples (68.57%) fell within the BP-recommended range (95%–105%), six showed the content of PGL below the range of 95%–105%. A control chart of the PGL contents following the BP limits is depicted in Figure 6. As shown, three samples (15C, 18C, and 19C) were found to be less than 80%. Additionally, three samples (20C, 29C, and 35C) were found to be below the lower limit (95%). Furthermore, five samples (1C, 3C, 5C, 10C, and 13C) exceeded the upper BP limit (105%). These results indicate that approximately one-third of the seized capsule samples did not satisfy the acceptable quality standard.

**TABLE 5:** UPLC-PDA analysis of seized pregabalin capsule samples. ND: not determined.

Capsule no.	Labelled capsule potency (mg)	Measured PGL concentration (mg/mg powder)	Total PGL content (mg) per capsule (mg/ capsule)	Assay of PGL%
1C	300	0.96	347.45	**115.82**
2C	150	0.65	148.28	98.85
3C	300	1.03	337.73	**112.58**
4C	150	0.75	154.15	102.77
5C	75	0.77	80.06	**106.74**
6C	75	0.80	75.13	100.17
7C	150	0.78	157.36	104.91
8C	150	0.78	155.53	103.69
9C	150	0.78	155.86	103.91
10C	75	0.84	81.57	**108.76**
11C	150	0.79	154.48	102.99
12C	150	0.70	150.60	100.40
13C	50	0.42	52.53	**105.06**
14C	300	1.02	300.22	100.07
15C	300	ND	ND	**ND**
16C	150	0.67	152.24	101.49
17C	150	0.68	155.49	103.66
18C	300	0.31	89.26	**29.75**
19C	150	0.35	77.96	**51.97**
20C	300	0.87	283.47	**94.49**
21C	300	0.94	301.95	100.65
22C	300	0.91	292.68	97.56
23C	75	0.57	72.83	97.11
24C	300	0.74	288.59	96.20
25C	300	0.77	310.10	103.37
26C	150	0.76	151.96	101.30
27C	150	0.77	154.77	103.18
28C	150	0.76	149.65	99.77
29C	50	0.28	46.34	**92.69**
30C	150	0.79	155.88	103.92
31C	300	0.78	309.90	103.30
32C	150	0.76	151.52	101.01
33C	300	0.94	300.46	100.15
34C	150	0.69	151.32	100.88
35C	300	0.60	280.75	**93.58**

Bold values indicate the seized capsule samples that did not satisfy the acceptable quality standard.

### 3.5 Analysis by LC-MS/MS

The UPLC-PDA method showed significant improvements over HPLC, reducing analysis time by 49.4% (from 17.0 to 8.6 min) and solvent waste by 74.8% (from 27.02 to 6.80 mL per sample). This was achieved through optimized parameters, including lowering the flow rate (from 1.0 to 0.5 mL/min) and decreasing the injection volume (from 20 to 3 μL), while doubling sample throughput from 2 to 4 samples per hour compared to HPLC.

This UPLC-PDA method achieved enhanced selectivity through its optimized chromatographic conditions. Importantly, the use of a 1.8 µm particle C18 column (50 × 2.1 mm) at 40°C improved peak sharpness and resolution compared to conventional HPLC, reducing peak widths and reducing co-elution risks. The method’s selectivity was enhanced by the buffered mobile phase (minimizing peak tailing) and 210 nm detection (avoiding matrix interference), while the small particle size (1.8 µm) and low injection volume (3 µL) improved peak symmetry and resolution. Despite the high efficiency of UPLC, challenges in impurity retention were addressed through gradient optimization, ensuring that early-eluting polar compounds (hydrophobic interactions) were resolved from the solvent front, while late-eluting nonpolar compounds were efficiently eluted within the 8.6 min runtime.

This approach not only improved separation efficiency but also met forensic requirements for rapid analysis and aligned with green chemistry principles by reducing solvent consumption while maintaining compliance with pharmacopeial standards.

### 3.6 Analysis by LC-MS/MS

LC-MS/MS analysis was performed to investigate the seized capsules and powder samples to identify any potential adulterants. The instrument was operated in multiple reaction monitoring (MRM) mode for targeted quantitative analysis. In the MRM mode, precursor and product ions are selected for each target analyte to achieve high selectivity and sensitivity.

Based on the molecular structures and fragmentation patterns, precursor and product ion transitions were selected for PGL and other common adulterants. These transitions were monitored in the mass spectrometer to detect and quantify analytes, if present, in the samples. Retention times and optimal collision energies were also determined from the analysis of standard solutions to allow the identification of peaks in the sample runs.

When sample extracts were injected into the LC column and eluted into the mass spectrometer, the transitions of PGL and monitored adulterants were automatically detected and peaks were produced in the chromatograms. By comparing retention times and peak areas to known standards, PGL, along with multiple adulterants in several seized capsule and powder samples, were distinguishable.

LC-MS/MS analysis revealed distinct fragmentation patterns characteristic of each analyte under optimized collision-induced dissociation (CID) conditions, validated against reference standards and published fragmentation patterns.

**FIGURE 7:**
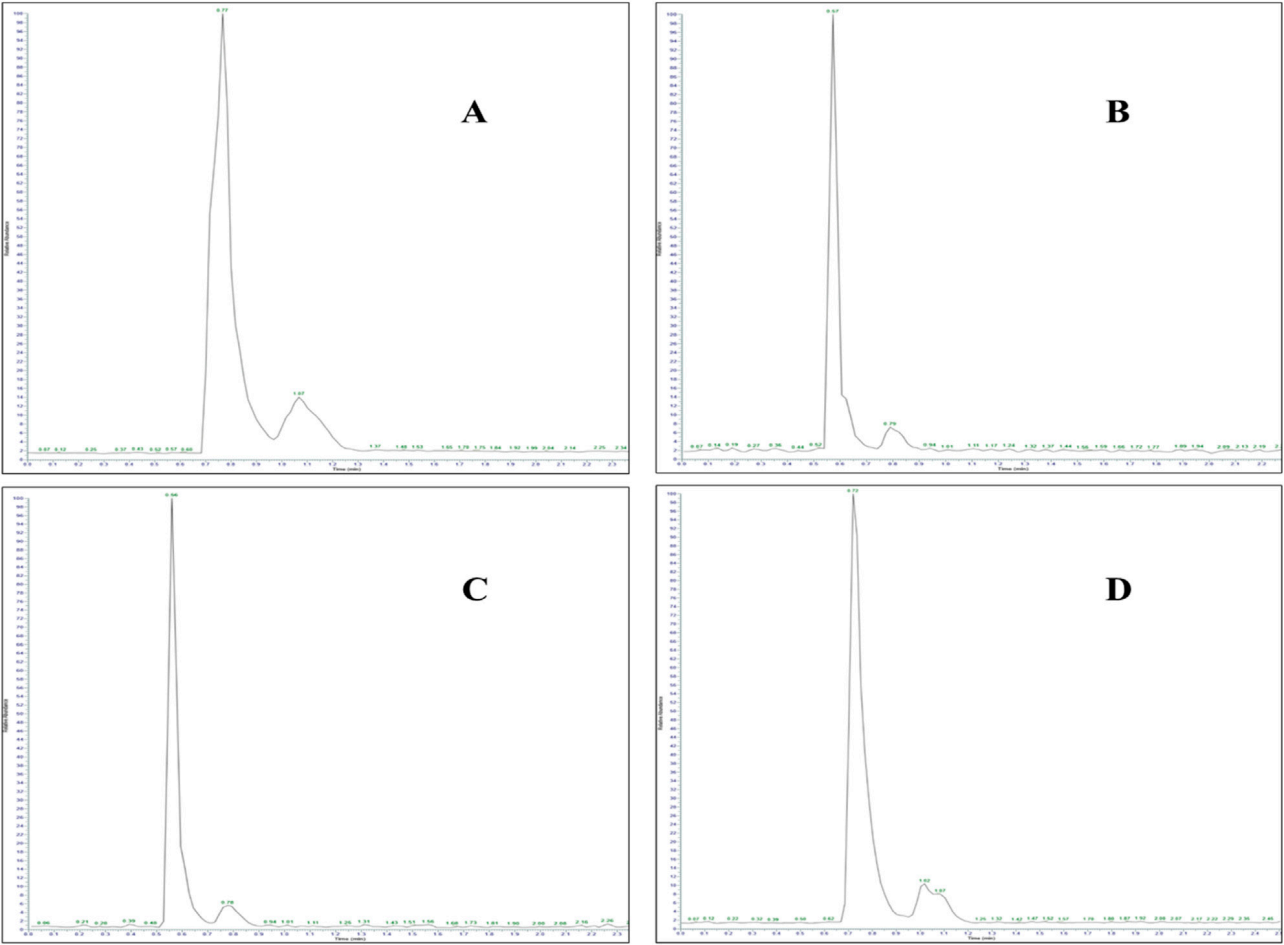
LC-MS/MS chromatogram of adulterated seized capsule sample containing **(A)** paracetamol (Rt: 0.77 min), **(B)** gabapentin (Rt: 0.57 min), **(C)** codeine (Rt: 0.56 min), and **(D)** pregabalin (Rt: 0.72 min).

**FIGURE 8:**
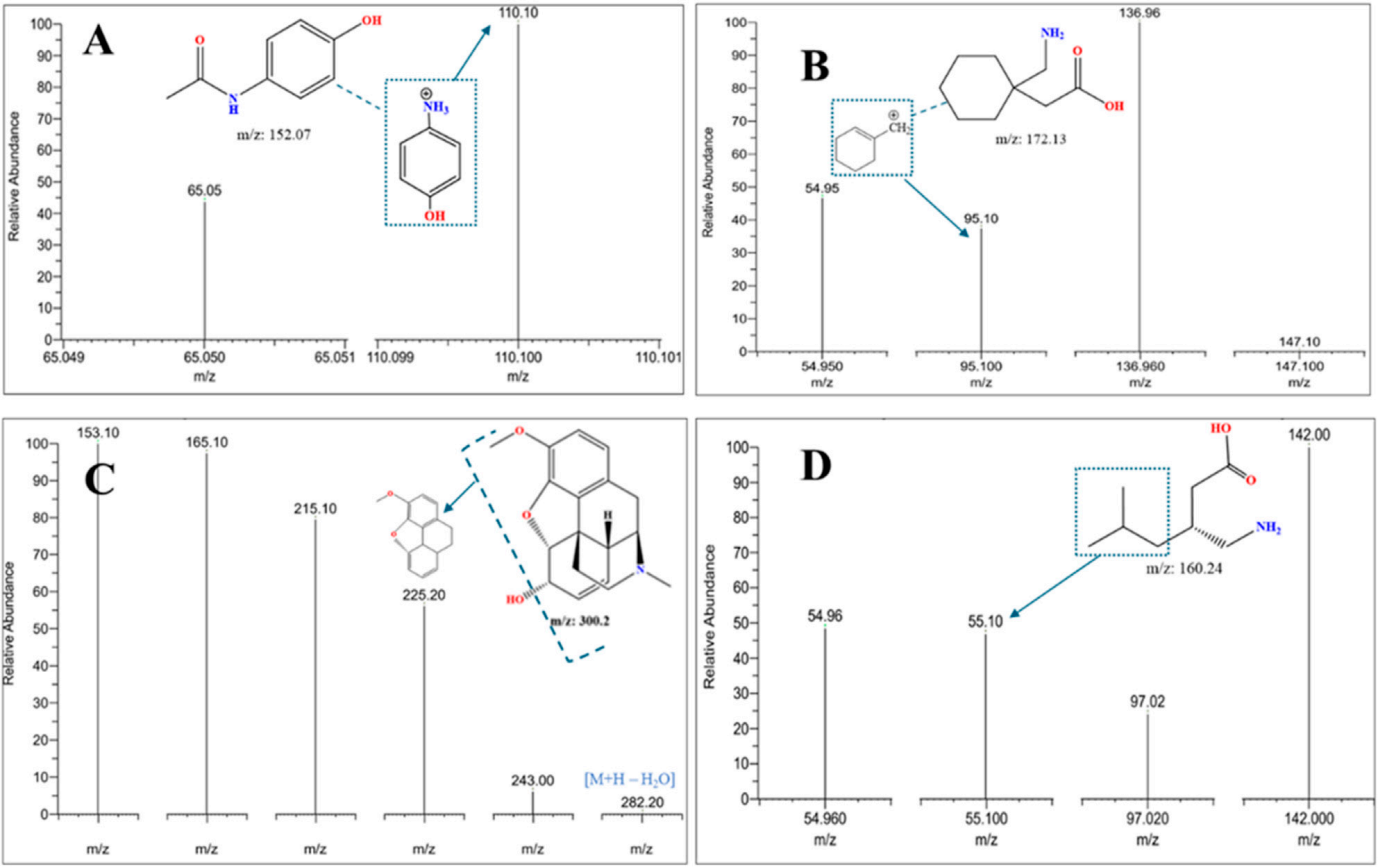
LC-MS/MS MRM mass spectra were acquired in positive ionization mode of fragmentation for an adulterated seized capsule sample containing **(A)** paracetamol, **(B)** gabapentin, **(C)** codeine, and **(D)** pregabalin, confirming the identity of co-formulated substances (paracetamol, gabapentin, codeine) in seized samples through MRM mass transitions.

The analysis revealed the presence of codeine, paracetamol, and gabapentin in various capsule samples (Figures 7, 8). This result confirmed adulteration in clandestine samples. As shown in Figure 8 and Table 6, the protonated molecular ion of paracetamol ([M + H]^+^ m/z: 152.1) was subjected to fragmentation, resulting in two diagnostic product ions when collision energies of 25 and 42eV were employed. The quantifier ion at m/z 110.1, arising from the elimination of the acetyl moiety (-COCH_3_), and the qualifier ion at m/z 65.05, corresponding to the phenyl ring fragment. The mass spectral behaviour of gabapentin was characterized by its protonated molecular at m/z 172.09, generating product ions at m/z 147.1 due to the loss of NH_3_, m/z 136.96 (sequential elimination of NH_3_ and CH_2_), and characteristic cyclohexyl-derived fragments observed included at m/z 95.1 and 54.95 with collision energies of 22 eV. On the other hand, codeine exhibited the most complex fragmentation profile, with its precursor ion [M + H]^+^ at m/z 300.2 yielding multiple structurally significant transitions: m/z 282.2 due to the dehydration process, m/z 243 resulting from combined losses of CH_3_OH and H_2_O, m/z 225.2 related to morphinan ring fragment, and diagnostic phenanthrene-derived ions at m/z 165.1 and 153.1 using collision energies ranging from 23 to 50eV. Together with PGL, which demonstrated a molecular ion [M + H]^+^ at m/z 160.09, exhibited sequential fragmentations to produce ions at m/z 142 (after H_2_O elimination), m/z 97.02 (resulting from consecutive losses of NH_3_ and CO_2_), and diagnostic alkyl fragments at m/z 55.1 and 54.96 using collision energies 10,14 and 22eV. In addition to the above-mentioned interpretation, the mass spectra of those compounds were compared with those of standard reference materials for confirmation.

**TABLE 6:** LC-MS/MS performance displays the precursor and product in (m/z) from the mass spectrum of the adulterated sample.

Compound	Precursor ion (m/z)	Product ion (m/z)	Collision energy (v)
Paracetamol	152.1	65.05	35
	152.1	110.1	20
Pregabalin	160.09	54.96	22
	160.09	55.1	22
	160.09	97.02	14
	160.09	142	10
Gabapentin	172.09	54.95	22
	172.09	95.1	22
	172.09	136.96	14
	172.09	147.1	16.2
Codeine	300.2	153.1	50
	300.2	165.1	46
	300.2	215.1	23
	300.2	225.2	25.4
	300.2	243	37
	300.2	282.2	37

It is noteworthy to mention here that the presence of codeine as an adulterant with PGL can possibly cause respiratory failure (Bykov et al., 2020). Many organizations, such as the FDA, CDC, and DEA declared warnings against this practice (Bockbrader et al., 2010). Moreover, the appearance of other materials, such as paracetamol (Figure 8) in some capsules, indicates unsatisfactory manufacturing practices that do not meet specified quality standards. Some capsules reduced amounts of PGL intended to deceive consumers. The assay results for these capsules showed concentrations much lower than expected. However, quantitative assessment revealed that other capsules contained high concentrations of PGL beyond expected levels, posing risks of toxicity to users.

Results demonstrated that most samples of PGL capsules and powders were either counterfeit drugs or exceeded limits as per international pharmacopoeias, i.e., BP and USP (British Pharmacopoeia, 2022; United States Pharmacopeia, 2023). In conclusion, this study revealed that all uncontrolled drugs and illicit drug trafficking pose life-threatening dangers to consumers not only because of the active substances themselves but also to mixtures containing other compounds, particularly those from unknown or untraceable clandestine sources.

Thus, through utilizing MRM-LC-MS/MS and targeted analyte transitions, seized drug samples were successfully profiled, impurities were detected, and adulteration trends were uncovered - providing valuable data on the illegal market and risks to public health.

The current UPLC-PDA method offers significant advantages over previous HPLC-UV assays for PGL quantification in terms of total run time and sample preparation. It achieves complete chromatographic separation within a substantially shorter run time of 8.6 min, compared to a previous study (Mutalabisin et al., 2021), which reported HPLC-UV method with longer run times (15 min) requiring derivatization with ninhydrin. This approach eliminates the need for derivatization, streamlining sample preparation and analysis time while minimizing potential sources of error associated with the derivatization process. The shorter run time enables higher throughput and increased efficiency, facilitating the analysis of a larger number of samples. The developed UPLC-PDA method demonstrates improved environmental sustainability compared to conventional HPLC-UV approaches through significant reductions in solvent consumption. The current method operates at an optimized flow rate of 0.5 mL/min, representing a 50% reduction from the previously reported HPLC-UV methods (1.0 mL/min) (Vinutha Ko et al., 2017; Khanage et al., 2014), while simultaneously decreasing the injection volume requirement. This enhancement in chromatographic efficiency aligns with the principles of green analytical chemistry by minimizing mobile phase usage and waste generation without compromising analytical performance. Additionally, the LC-MS/MS data further highlighted custom mixing of PGL with codeine, a trend noted in a previous study (Buttram and Kurtz, 2020) in illicit markets.

## 4 Conclusion

In this study, 40 seized samples (powder and capsule samples) were analysed using UPLC instrumentation. The capsules were traced to determine their manufacturing origin. Additionally, the obtained raw materials packaged in capsules were examined to understand their intended marketing. The content of these capsules had been adulterated, with adulterated samples showing concentrations inconsistent with registered amounts at the regulatory bodies.

Various capsule samples contained mixtures of PGL and codeine, a derivative of morphine that can cause serious respiratory failure. Moreover, results included either low drug amounts intended to mislead consumers or high concentrations beyond expected levels, posing toxicity risks. Overall, the analytical examination revealed poor practices in smuggled pharmaceuticals threatening public health and safety. These current chromatographic methods demonstrated significant performance characteristics for detecting PGL in seized samples and showed reliable quantitative and qualitative analysis. The analytical capabilities allowed simultaneous detection of multiple compounds, including pregabalin, codeine, gabapentin and paracetamol. This multi-compound detection feature proved crucial in identifying adulterants and determining sample authenticity. The chromatographic separation, combined with LCMS confirmation, provided accurate and dependable results for both powder and capsule formulations, making it an ideal tool for forensic analysis and quality control purposes.

## Data Availability

The original contributions presented in the study are included in the article/supplementary material, further inquiries can be directed to the corresponding author.
