# Correction: Investigation of illicit pregabalin in seized samples from Saudi Arabia

**DOI:** 10.3389/fchem.2025.1674670

**Published:** 2025-08-07

**Authors:** Frontiers Production Office

**Affiliations:** Frontiers Media SA, Lausanne, Switzerland

**Keywords:** pregabalin, forensic analysis, abused drugs, seized samples, counterfeit drugs, adulterants, ultra-performance liquid chromatography, tandem mass spectrometry

Authors Haitham Al-Hamoud and Yahya F. Jamous were erroneously assigned to the affiliation “General Directorate of Narcotics Control, Ministry of Interior (MOI), Riyadh, Saudi Arabia”. The affiliation “Institute of Refining and Petrochemical Technologies, Energy and Industry Sector, King Abdulaziz City for Science and Technology (KACST), Riyadh, Saudi Arabia” was omitted for author Haitham Al-Hamoud. The affiliation “Institute of Health Technologies and Preventive Medicine—Health Sector, King Abdulaziz City for Science and Technology (KACST), Riyadh, Saudi Arabia” was omitted for author Yahya F. Jamous. These affiliations have now been added.

The original article has been updated.

